# Determining the Role of UTP-Glucose-1-Phosphate Uridylyltransferase (GalU) in Improving the Resistance of *Lactobacillus acidophilus* NCFM to Freeze-Drying

**DOI:** 10.3390/foods11121719

**Published:** 2022-06-12

**Authors:** Zhidan Zeng, Xiaoqun Zeng, Yuxing Guo, Zhen Wu, Zhendong Cai, Daodong Pan

**Affiliations:** 1 State Key Laboratory for Managing Biotic and Chemical Threats to the Quality and Safety of Agro-Products, Ningbo 315211, China; zengzhidan@genomics.cn (Z.Z.); guoyuxing1981@163.com (Y.G.); wuzhen@nbu.edu.cn (Z.W.); caizhendong@nbu.edu.cn (Z.C.); pandaodong@nbu.edu.cn (D.P.); 2 Key Laboratory of Animal Protein Food Processing Technology of Zhejiang Province, College of Food and Pharmaceutical Sciences, Ningbo University, Ningbo 315800, China; 3 School of Food Science and Pharmaceutical Engineering, Nanjing Normal University, Nanjing 210097, China

**Keywords:** *galU*, *Lactobacillus acidophilus*, gene knockout, gene expression, freeze-drying, metabolic pathway

## Abstract

*Lactobacillus acidophilus* NCFM is widely used in the fermentation industry; using it as a freeze-dried powder can greatly reduce the costs associated with packaging and transportation, and even prolong the storage period. Previously published research has reported that the expression of *galU* (EC: 2.7.7.9) is significantly increased as a result of freezing and drying. Herein, we aimed to explore how *galU* plays an important role in improving the resistance of *Lactobacillus acidophilus* NCFM to freeze-drying. For this study, *galU* was first knocked out and then re-expressed in *L. acidophilus* NCFM to functionally characterize its role in the pertinent metabolic pathways. The knockout strain Δ*galU* showed lactose/galactose deficiency and displayed irregular cell morphology, shortened cell length, thin and rough capsules, and abnormal cell division, and the progeny could not be separated. In the re-expression strain p*galU*, these inhibited pathways were restored; moreover, the p*galU* cells showed a strengthened cell wall and capsule, which enhanced their resistance to adverse environments. The p*galU* cells showed GalU activity that was 229% higher than that shown by the wild-type strain, and the freeze-drying survival rate was 84%, this being 4.7 times higher than that of the wild-type strain. To summarize, expression of the *galU* gene can significantly enhance gene expression in galactose metabolic pathway and make the strain form a stronger cell wall and cell capsule and enhance the resistance of the bacteria to an adverse external environment, to improve the freeze-drying survival rate of *L. acidophilus* NCFM.

## 1. Introduction

*Lactobacillus acidophilus* NCFM is one of the most widely used probiotic species in the fermentation industry [1,2]. It has been reported to improve intestinal flora composition [3], regulate metabolism levels [4], enhance immune function, and also prevent cancer [5]. Using the freeze-drying method to obtain *L. acidophilus* powder can greatly reduce the costs associated with packaging and transportation, and even prolong the storage period [6]. However, upon exposure to stress in the form of freezing and drying, the survival rate of *L. acidophilus* is adversely impacted, which is not conducive to the industrial production of *L. acidophilus* powder [7]. To improve the survival rate of freeze-dried strains, methods such as improving culture conditions [8], adding sugars to protective solutions [9], and optimizing the freeze-drying parameters [10] have been employed, but the survival rate of strains continues to remain low in mass-production environments.

Upon freezing and drying *L. acidophilus* NCMF, the mRNA transcription of UTP-glucose-1-phosphate uridylyltransferase (GalU, EC: 2.7.7.9, encoded by *galU*) has been reported to significantly increase [11]. Furthermore, it has been speculated that the survival rate of freeze-dried *L. acidophilus* NCFM can be improved via *galU* (903 nt, Gene ID: 3253049). Therefore, in this study, we tried to knock out the *galU* gene and then re-express it to establish whether its lactose metabolism was affected. The effect of the *galU* gene on *L. acidophilus* NCFM in freeze-drying was evaluated by observing the morphological changes, growth curves, and freeze-dried survival rates after knockout and re-expression.

The *galU* gene plays a key role in glycogen synthesis in animals [12] and regulates the conversion process between starch and polysaccharides in plants [13]. In *Streptococcus pneumoniae*, *galU* directly affects growth, adhesion, in vitro phagocytosis, and in vivo pathogenicity [14]. Moreover, in uropathogenic *Escherichia coli*, the mutation of *galU* has been observed to result in the loss of the O-polysaccharide sidechain of lipopolysaccharides, consequently affecting the post-translational modification of proteins [15]. However, to date, only a few studies have explored how *galU* improves the resistance of *L. acidophilus* NCFM to freeze-drying. Therefore, in this study, transcriptomes are used to further analyze the differences among *L. acidophilus* NCFM and its knockout and re-expression offspring.

## 2. Materials and Methods

### 2.1. Strains and Growth Conditions

The bacterial strains and plasmids are listed in Table 1. The LA strain was statically cultured in Man–Rogosa–Sharpe (MRS) medium at 37 °C, with 2% inoculation [16]. For knockout plasmid preparation, *E. coli* strain DH10BT1 carrying pK18mobsacB was cultured in 50 mL Luria–Bertani (LB) medium containing 50 µg/mL kanamycin, followed by incubation at 37 °C in a rotary shaker (150 rpm) for 18 h [17]. MRS medium, containing 5 µg/mL ampicillin, was used for screening positive clones harboring low-copy recombinant knockout vectors. SAMRS (MRS medium with 10% sucrose) medium was used for the negative screening of *galU*-deleted strains [18]. M17 medium, with lactose as the sole source of carbon, was used for identifying and screening the lactose-deficient strains [19]. GM17 medium (M17 medium with 5% glucose) was used to extract pNZ8149 and culture the lactose-deficient strains [20]. Then, 0.04% bromocresol violet was added to the M17 medium (BM17 medium), which served as an indicator (colonies appeared yellow) when lactose was fermented by *Lactobacillus* to produce acid [21].

### 2.2. Knockout of galU

The upstream and downstream homologous arms of *galU* and the gene responsible for ampicillin resistance (*amp*) in the pUC57 plasmid were linked using a CV19 One-Step Seamless Cloning kit (Aidlab Biotechnologies Co., Ltd., Beijing, China) to construct Knock, a target segment for *galU* knockout. The upstream and downstream homologous arms of *galU* were amplified using *galU*-1-F/R and *galU*-2-F/R primers, and *amp* was amplified using *amp*-F/R primers, with pUC57 serving as the template. The primer sequences are listed in Table 2.

After double-digestion with the restriction endonucleases *Bam*HI and *Pst*I, the linear Knock fragment and the pK18mobsacB vector were ligated (2:1 ratio) using the T4 DNA ligase, followed by incubation at 37 °C overnight [22]. The product was transferred into *E. coli* Trans-T1 cells and positive clones were verified by performing PCR with *galU*-1-F and *galU*-2-R primers. The DNA sequence of positive clones with a 99.9% matching rate was named Knock-pK18mobsacB (i.e., the recombinant knockout vector).

Subsequently, Knock-pK18mobsacB was electro-transformed into competent *L. acidophilus* cells (1.2 kV, 25 μF, 200 Ω, 5.1 ms pulses) using a gene pulser transfection apparatus (Xinyi-2E, Ningbo Xinyi Co., Ltd., Ningbo, China). After recovery for 3 h in MRS broth, the bacterial solution was evenly spread onto an MRS medium plate containing 5 mg/mL ampicillin [23]. After incubation for 3 days, colonies were selected for expanded culture, and PCR was performed with *galU*-1-F and *galU*-2-R primers for validation. Positive bacterial cells harboring the target segment were spread onto a SAMRS-medium plate and allowed to grow for 3 days; colonies were then selected for validation via PCR. Positive strains with a matching rate of > 99.9% by sequencing were named Δ*galU* (i.e., the galU knockout strain). Using the wild-type strain, LA, three pairs of primers for *galU* (*galU*-4/5/6-F/R) were designated to confirm that *galU* was knocked out.

In order to verify whether the lactose metabolic pathway of Δ*galU* was knocked out, the LA and Δ*galU* strains were adjusted to OD_600_ 1.0 and then diluted 10^6^ times with sterile physiological saline; we then drew an S-shaped curve on a lactose plate to observe whether growth could be seen after culturing at 37 °C for 36 h.

### 2.3. Expression of galU in ΔgalU

*The galU* (903nt) gene was amplified using *galU*-7-F/R primers and LA-strain DNA as the template, and pNZ8149 from *L. lactis* was digested by incubation with *Nco*I and *Xba*I at 37 °C overnight [24]. The DNA was denatured at 94 °C for 2 min, annealed at 60 °C, and then extended at 72 °C for 1 min in 30 cycles for Polymerase Chain Reaction (PCR) amplification. The purified products were linked using a CV19-One Step Seamless Cloning kit (Aidlab Biotechnologies Co.,Ltd., Beijing of Chian) to obtain the recombinant expression plasmid pNZ8149-*galU*, which was transfected into competent ∆*galU* cells via electroporation [25]. After incubation in MRS broth for 3 h, positive clones were screened on BM17 medium plates and identified via PCR with *galU*-8-F/R primers. The Δ*galU* strain harboring pNZ8149-*galU* with a 99% matching rate by sequencing was named p*galU* (i.e., the *galU* re-expression strain). LA, Δ*galU*, and p*galU* strains were placed on the S line of a BM17 medium plate, and colony morphology was observed after incubation at 37 °C for 36 h. The three strains, p*galU*, LA, and Δ*galU,* were expanded in MRS broth for 18 h and then collected; the sediment was then resuspended with 2 mL of sterile saline, 100 µL of lactose (purple) and galactose (green) was added to the fermentation tube, and incubation at 37 °C for more than 18 h was used to observe the color change. If the strain could ferment lactose or galactose to produce acid, the solution turned yellow.

### 2.4. Determination of GalU Activity

Growth curves were constructed for the LA, Δ*galU*, and p*galU* strains grown in an MRS medium with 1% of inoculation; we measured the OD_600_ value every 2 h and plotted the measured OD_600_ value and corresponding culture time, then collected the stable stage of the strain according to the growth curve, which was followed by centrifugation of 50 mL bacterial cell suspension at 5000× *g*. The cells were then washed with 0.1 M phosphate-buffered saline, resuspended, and lysed using an ultrasonic cell disrupter (Scientz-IID, Scientz Biotechnology Co., Ltd., Ningbo, China). Cell lysis was performed at 300 W, with 100 s pulses and 3 s pauses, on an ice bath to prevent protein denaturation [26]. Subsequently, a 2 mL sample of lysed cells was centrifuged at 12,000× *g* for 10 min at 4 °C. The supernatant was transferred to a new centrifuge tube, and the remaining precipitate was dissolved in 2 mL denaturant buffer (8 M urea, 100 mM NaH_2_PO_4_, 10 mM Tris-HCl, pH 8.0). The total protein content was determined with a bicinchoninic acid kit for protein determination (Sigma-Aldrich, Shanghai, China). According to the determined results, the total protein concentration of each copy was adjusted to 0.1 mg/mL [27], and the enzyme activity of GalU (34.46 kDa) was detected with an ELISA Kit (Shanghai Keshun Biotechnology Co., Ltd., Shanghai, China) according to the instructions: we added 0.05 mL of sample to reaction wells that had been coated with GalU antibodies, incubated them at 37 °C for 1 h and then washed them, establishing the blank and standard curves at the same time. Each well was washed after adding 0.05 mL of microplate antibody and then incubated at 37 °C for 1 h. We then added 0.1 mL of TMB substrate solution and incubated the wells at 37 °C for 30 min; finally, we added 0 05 mL of 2 M sulfuric acid to terminate the reaction. Immediately afterward, we determined the absorbance value at 450 nm with a microplate reader and calculated the GalU activity, according to the standard curve.

### 2.5. Effect of Freeze-Drying on Bacterial Survival Rate

Growth curves were used to determine the effects of freeze-drying on bacterial survival rate. The strains were cultured to the end of the stationary phase (OD_600_ of around 1.2), followed by centrifugation of 50 mL bacterial cell suspension at 5000× *g* for 10 min, the precipitates were collected and frozen overnight at −80 °C, and then dried in an Alpha 1-4 LD Plus freeze-dryer (Christ Goema, Germany) for 24 h at −49 °C and 9 Pa. After the cells were freeze-dried for 24 h, they were rehydrated immediately after being taken out of the freeze dryer (at room temperature) without storage. At the same time, pre-frozen and freeze-dried samples were placed in 50 mL sterile tubes. We then took 1 mL of bacterial solution before and after lyophilization (adding 50 mL sterile saline for re-dissolution), diluted it by 10^3^, 10^4^, 10^5^, 10^6^, 10^7^, and 10^8^ times, and coated the plates, with three parallels in each group. Plate colony-counting was performed after 3 days of incubation, and the freeze-drying survival rate was calculated as the number of live bacteria after lyophilization, divided by the number of live bacteria before lyophilization × 100% [28].

### 2.6. Transmission Electron Microscopy (TEM) to Assess the Cell Structure

To obtain the bacterial cells, the LA, Δ*galU*, and p*galU* strains were centrifuged at 3000× *g* for 10 min at 4 °C. After washing twice with 0.1 M phosphate-buffered saline, the cells were fixed in 2.5% glutaraldehyde solution for > 12 h at 4 °C. The samples were immersed in 0.1 M phosphate-buffered saline thrice for 15 min each time, and then fixed in 1% osmium acid, followed by incubation for 1–2 h in a dark room [29]. After three times washes with 30%, 50%, 70%, and 90% alcohol for 15 min respectively, the samples were treated three times with 90% acetone and anhydrous acetone for 15 min each time. After overnight incubation with an embedding agent, fresh embedding agent was added, and polymerization was allowed to proceed at 37 °C for 12 h, then the samples were dried at 60 °C for 36 h. Subsequently, the samples were sliced into 50–60 nm slices using an LKB-1 ultrathin slicer. The cells were observed using an H-800 transmission electron microscope (Hitachi, Tokyo, Japan) after double-staining with 3% uranium acetate.

### 2.7. Transcriptome Sequencing

LA, Δ*galU*, and p*galU* strains were cultured in an MRS medium to an OD_600_ of around 1.2, and total RNA was then extracted using a kit (Qubit 4.0). After rRNA removal, oligo-(dT) magnetic beads were added for mRNA enrichment, and short mRNA fragments were obtained. After synthesizing, modifying, purifying, and segmenting the fragments, they were sequenced on an Illumina HiSeq 2000. The NGS QC software was used to filter and count the reads, in order to identify the different genes and analyze the metabolic pathways [30].

## 3. Results

### 3.1. Acquisition of the galU Knockout Strain ΔgalU

**Figure 1:**
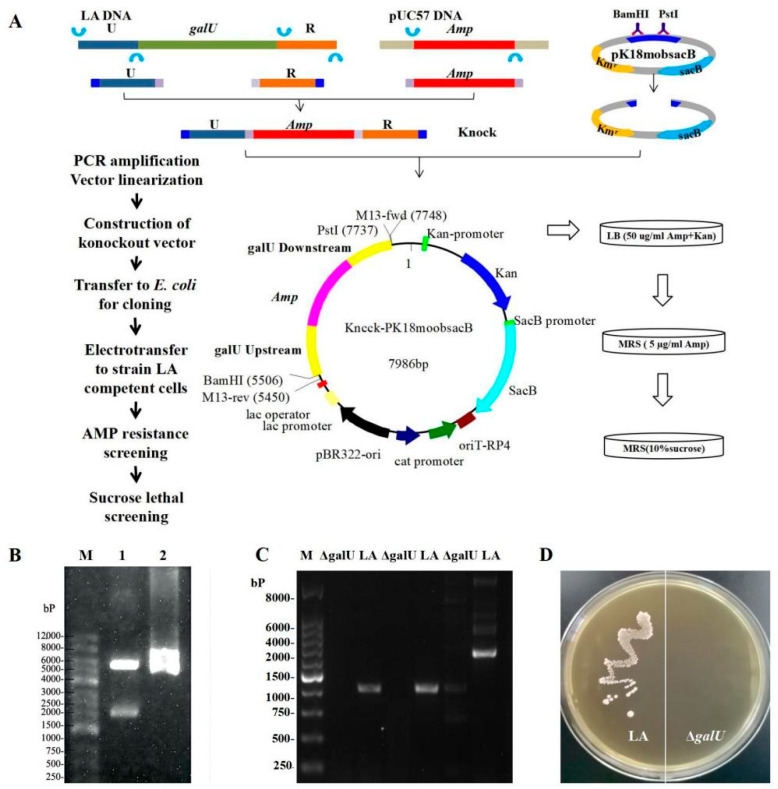
Schematics and results for the knockout of the *galU* gene. (**A**) Schematics for the knockout of the *galU* gene. (**B**) BamHI-PstI double-digestion map. Lane M, Marker 12,000 bp; Lane 1, fragments of the digested plasmid, Lane2, recombinant plasmid Knock-pK18mobsacB. (**C**) PCR amplification of the knockout strain Δ*galU* and original LA strain. Lane M, Marker 8000 bp; Δ*galU*, PCR amplification of the knockout strain Δ*galU*; LA, PCR amplification of the original LA strain. (**D**) The growth of the original strain LA and knockout strain Δ*galU* on an M17 plate, with lactose as the sole glycogen. The left side of the plate is the original strain LA, which can form normal colonies. The right side of the plate is the knockout strain, Δ*galU*, without colony formation.

The 2180 bp Knock target segment was synthesized from the 662 bp upstream and 592 bp downstream homologous arms of *galU* and the 974 bp *amp* gene, followed by ligation in pK18mobsacB to obtain Knock-pK18mobsacB (Figure 1A); BamHI–PstI double-digestion was then performed for validation (Figure 1B). After introducing Knock-pk18mobsacb into the LA strain, the knockout strain Δ*galU* was obtained, as verified through sequencing. In the case of the wild-type strain LA, PCR using three pairs of *galU* primers (*galU*-4/5/6-F/R) generated the corresponding bands, but no amplicons were observed in the case of Δ*galU* (Figure 1C), indicating that the *galU* in Δ*galU* had been successfully knocked out. As shown in Figure 1D, the LA strain could grow on an M17 agar plate, but Δ*galU* could not grow on an M17 agar plate, signifying that Δ*galU* was unable to decompose lactose into glucose so as to maintain growth.

### 3.2. Acquisition of the galU Re-Expression Strain pgalU

**Figure 2:**
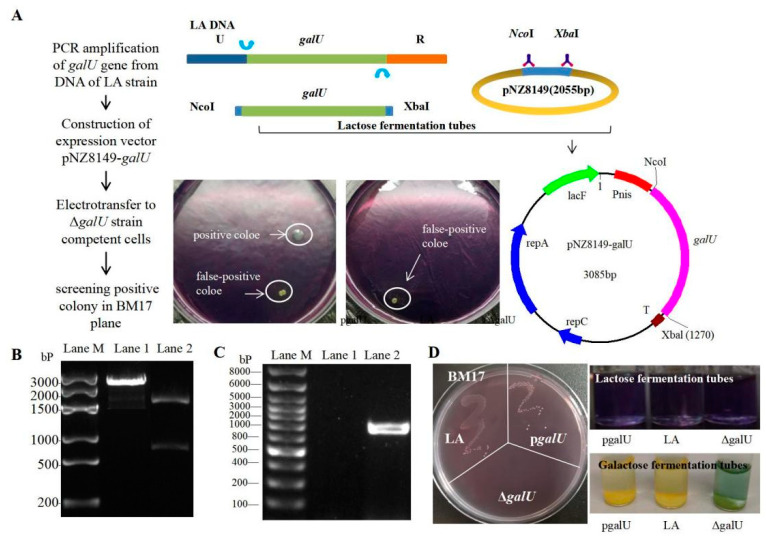
Schematics and results of preparation of the p*galU* complementation strain. (**A**) Schematics for preparation of the overexpression strain, p*galU*. (**B**)Electrophoresis of recombinant expression plasmid pNZ8149-*galU*. Lane M, Marker 3000 bp; Lane 1, fragments of the digested plasmid; Lane 2, plasmid pNZ8149-*galU* digested by NcoI-XbaI restriction endonucleases. (**C**) PCR validation of a positive p*galU* strain, screened on a BM17 plate. Lane M, Marker 8000 bp; Lane 1, a pseudo-positive clone; Lane 2, positive p*galU* strain. (**D**) BM17 plate colony (**left**) and the lactose/galactose fermentation (**right**) of the LA, Δ*galU,* and p*galU* strains.

Considering the fact that the knockout strain Δ*galU* showed lactose deficiency, we concluded that the food-grade expression vector pNZ8149 could be used for *galU* expression. Next, pNZ8149 and *galU* (obtained by PCR amplification of DNA obtained from the LA strain, Figure 2B) were recombined to obtain the food-grade expression plasmid, pNZ8149-*galU* (verified by NcoI–XbaI double digestion, Figure 2C), which was then introduced into Δ*galU*, and the positive clones were screened on BM17 agar. As is evident from Figure 2A, Δ*galU* showed growth on BM17 agar only upon the successful integration of pNZ8149-*galU*. There was no colony of Δ*galU* found on the BM17 plate, while the LA and p*galU* strains were similar (Figure 2D, left). The lactose fermentation tubes (purple) of the p*galU*, LA, and Δ*galU* strains have not changed color, indicating that none of the three strains can directly use lactose fermentation to produce acid. The galactose fermentation tubes of the p*galU* and LA strains have become yellow, while the Δ*galU* strain has not (Figure 2D, right), indicating that the galactose fermentation pathway of the Δ*galU* strain has been blocked and has been fixed in the p*galU* strains.

### 3.3. GalU Activity of the LA, ΔgalU, and pgalU Strains

**Figure 3:**
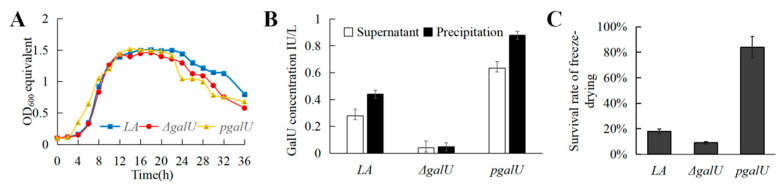
Growth curves, GalU enzyme activity and freeze-drying survival rate for strains LA, Δ*galU* and *pgalU.* (**A**) Growth curves of strains LA, Δ*galU* and p*galU*. (**B**) GalU enzyme activity in strains LA, Δ*galU*, and p*galU*. (**C**) The freeze-drying survival rate for strains LA, Δ*galU,* and p*galU*.

The three strains entered the logarithmic phase of growth from around 4 h onward and the stable phase at 8 h; the maximum OD_600_ value stabilized at 1.45*–*1.50, then gradually declined after 20 h (Figure 3A). According to the standard curve of GalU activity determination, the GalU content in the LA, Δ*galU,* and p*galU* strains was evaluated (Figure 3B). The knockout strain Δ*galU* showed almost no GalU activity, while the re-expression strain p*galU* showed GalU activity that was 229% higher than that of the wild-type strain, with an increased amount of precipitate.

### 3.4. Effect of galU on Freeze-Drying Survival Rate

In the freeze-drying experiment, the survival rate of Δ*galU* was only 9%, while that of p*galU* was 84%, which was 4.7 times that of LA (17.9%; Figure 3C). These results indicated that *galU* expression substantially contributes to increasing the survival rate of freeze-dried strains; this may be related to the strengthening of the cell wall and capsule.

### 3.5. TEM of LA, ΔgalU, and pgalU Strains

**Figure 4:**
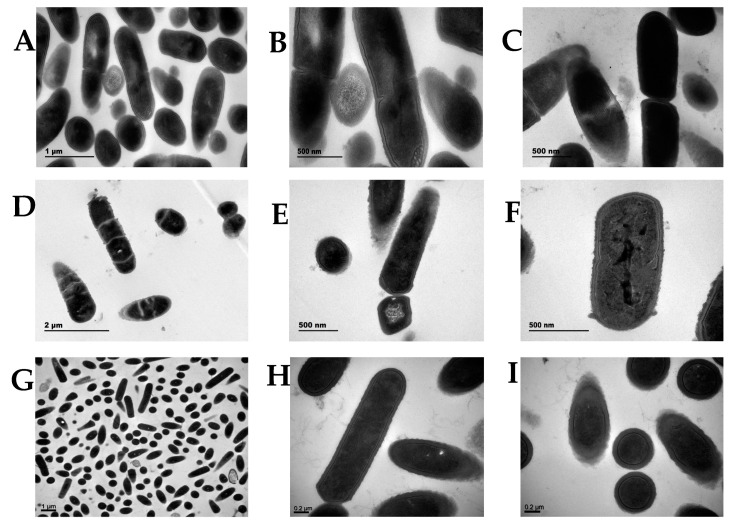
Colony morphology and microscopic morphology of the LA, Δ*galU,* and p*galU* strains. (**A**–**C**) Transmission electron microscopy of strain LA. (**D**–**F**) The *galU* gene knockout strain, Δ*galU*. (**G**–**I**) The *galU* gene expression strain, p*galU*.

TEM revealed that the wild-type LA cells (Figure 4A–C) were short, rod-shaped, and 1 μm long. A dense capsule was present around them, conferring higher resistance to adverse environments. In contrast, Δ*galU* cells showed obvious changes in their cell structure (Figure 4D–F); the cells were irregular and the capsule was thin and rough. Although Δ*galU* cells could continue to replicate and divide, the progeny could not be separated and only shared the original cell shell. In the TEM experiment, the first-generation knockout strain Δ*galU* that had just been selected was used. The growth of the first-generation Δ*galU* monoclonal strain was very slow and the survival rate was very low. According to the uniform treatment of the strains in the pre-TEM stage, the monoclonal strains were picked out and incubated for the same time, then centrifuged. After the cells were collected, they were fixed with formaldehyde. It can be observed that the precipitation of the knockout bacteria was significantly less than that of the wild-type strains. After many iterations, the growth status of Δ*galU* gradually became consistent with that of the wild type. Furthermore, the re-expression strain p*galU* showed normal morphology and cell division; the p*galU* cells showed significant growth and the capsule appeared thick (Figure 4G–I).

### 3.6. Regulation of Metabolic Pathways by galU

**Figure 5:**
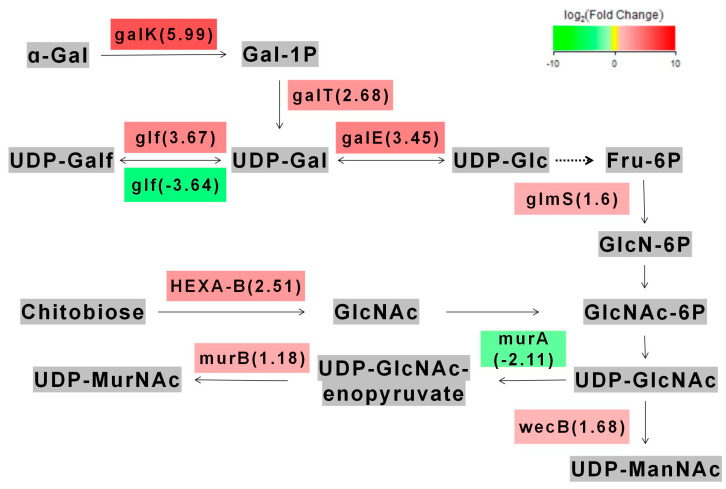
The main metabolic pathways affected by galU gene.

We found that 410 genes were upregulated and 1196 genes were downregulated in the metabolic pathways of *L. acidophilus* after *galU* knocked out. In the amino sugar metabolism pathway, we found that the part of the *galU* genes we edited were mainly involved in the regulation of galactose metabolism (here we only compared the start strain LA with the re-expression strain p*galU*, because the knockout strain Δ*galU* could not be cultured in lactose) (Figure 5). Through gene enrichment analysis, eight genes with the highest expression difference were identified: galactokinase (galK), UDPglucose--hexose-1-phosphate uridylyltransferase (galT), UDP-glucose 4-epimerase (galE), UDP-galactopyranose mutase (glf), glutamine---fructose-6-phosphate transaminase (glmS), UDP-N-acetylglucosamine 2-epimerase (wecB), UDP-N-acetylmuramate dehydrogenase (murB), hexosaminidase (HEXA_B). Their Q value values were <0.01, and the degree of enrichment was very significant. These genes are related to the transformation of galactose into UDP-ManNAc. This result could be attributed to the recovery of galactose metabolism and improvement of freeze-drying resistance in p*galU*.

## 4. Discussion

We herein investigated the mechanisms responsible for improving freeze-drying resistance by first knocking out and then re-expressing galU in L. acidophilus NCFM. The knockout strain ΔgalU showed lactose deficiency, irregular cell morphology, abnormal cell division, and thin and rough capsule; moreover, lactose metabolism ability was lost. After galU was re-expressed, galactose metabolism ability was restored and genes are related to the transformation of galactose into UDP-ManNAc showed higher expression levels, and the cell wall and capsule became thicker. Our previous work found that mannose as antifreeze factors can improve the survival rate of L. acidophilus after freeze-drying, and the enzyme activities detection also showed the activity of glycosyltransferases such as GalU had significant difference in adding mannose as antifreeze factors [31]. Therefore, we supposed and verified that the galU gene as an important regulatory site for L. acidophilus for resisting freeze-drying and UDP-ManNAc-related amino sugars can be used as antifreeze factors for L. acidophilus.

During sample preparation for transcriptome sequencing, MRS medium (contains glucose, not lactose, as the main source of carbon) was used to culture all three strains (LA, ΔgalU and pgalU), that may underestimate the espressions of genes in galactose metabolism pathway. In L. acidophilus NCFM, lactose can be hydrolyzed to glucose and galactose under the action of β-galactosidase after being transported into the cell [32,33]. After β-galactosidase binds to lactose, glucose is first released [34,35]. Therefore, lactose metabolism in ΔgalU must be inhibited before glucose is released in the reaction of galactosidase and lactose. We speculate that galU knockout may resulted in ΔgalU losing its ability to hydrolyze lactose.

In this study, one of the reasons for using pNZ8149 was that it is a food-grade expression vector [36], making it ideal for food research and development [37]. The other reason was that NZ3900, the standard host strain of pNZ8149, is also a lactose-deficient strain [38,39]. The NZ3900 strain requires the presence of lacF/repA/C in pNZ8149 to ensure a functional lactose metabolism pathway [40]. Theoretically, both pNZ8149 and *galU* can ensure the growth of the knockout strain Δ*galU* on the M17 plate, but the experimental results showed that pNZ8149 makes the colony yellow, while *galU* makes the colony white. In this manner, we could distinguish whether the recovery of lactose metabolism in the different strains was affected by pNZ8149 or *galU*. The reason why the colonies appear to have different colors needs further exploration. What is more, it is hard to understand why gene expression often needs to be induced by inducers [41,42], but p*galU* could efficiently express the *galU* gene in a lactose medium, even without the inducers, and the colonies formed were larger and moister. This might be attributed to two reasons: one is that lactose, as an inducer, is influenced by the lactose-specific element lacZ gene, leading to *galU* expression [43,44]; the other is that Δ*galU* is more inclined to transcribe DNA damage-repair genes, particularly when encountering adverse environments [45,46,47]. Thus, the *galU* expression in Δ*galU* was stronger. Further studies are warranted to explore the pertinent mechanisms.

In this paper, we show that the Δ*galU* strain can serve as an efficient expression system, with the expression vector containing *galU*. Using lactose agar, strains with positive expression can be screened, even in the absence of inducers in the growth medium. This screening method is simple and does not involve the use of antibiotics. We aim to further study the knockout strain Δ*galU* to validate its safety and expression mechanism, and we expect Δ*galU* to become a food-grade high-efficiency expression system of *L. acidophilus*.

## Figures and Tables

**Table 1:** The bacterial strains and plasmids used in this study.

Strain or Plasmid	Relevant Characteristic (s)	Source or Reference
*Strains*		
*Escherichia coli* Trans1 T1	F^−^φ80 (*lacZ*) ΔM15ΔlacX74*hsdR* (r_k_−, m_k_+) ΔrecA1398*end*A1*ton*A	TransGen Biotech
*Escherichia coli* DH10BT1	F^−^ mcrA Δ(mrr-hsdRMS-mcrBC) Φ80lacZΔM15ΔlacX74 recA1 endA1araD139Δ (ara, leu) 7697 *galU* galKλ- rpsL nupG tonA	Biovector NTCC
*Lactobacillus acidophilus* NCFM (LA)	Wild-type strain	ATCC
Δ*galU*	LA strain with *galU* deleted	This work
NZ3900	*lac*F^−^, *pep*N: *nis*R *nis*K	Biovector NTCC
p*galU*	Δ*galU* strain with plasmid pNZ8149-*galU*	This work
Plasmid		
pUC57	Ap^r^; *lac*Z/MCS; pMB11 *ori*	Biovector NTCC
pK18mobsacB	Km^r^; *lac*Z/MCS; pBR322 *ori*; *sac*B	Laboratory collection
Knock-PK18mobsaB	Km^r^; MCS (with BamHI and PstI); sacB	This work
pNZ8149	*lac*F; *nis*A; nisC; MCS	Biovector NTCC
pNZ8149-*galU*	*lac*F; *nis*A; *nis*C; MCS (with *Nco*I and *Xho*I)	This work

**Table 2:** The primers used in this study.

Primer	Sequence	Position in Chromosome
*galU*-1-F (LA)	5′-ggatccGCGAACAACTCTTTCACAA	610275
*galU*-1-R (LA)	5′-GAAATGTTGAATACTCATGATAACGCCAGCCAACCAA	610898
*galU*-2-F (LA)	5′-CTGATTAAGCATTGGTAATGGCTCGTCAAGTTGCTCT	612410
*galU*-2-R (LA)	5′-ggaattccCTGGCACCGTCAGTAAGAG	612957
*amp*-F (pUC57)	5′-TTGGTTGGCTGGCGTTATCATGAGTATTCAACATTTC	1650
*amp*-R (pUC57)	5′-AGAGCAACTTGACGAGCCATTACCAATGCTTAATCAG	2492
*galU*-4-F (LA)	5′-TCCATAACCGAGTAGGAGA	611061
*galU*-4-R (LA)	5′-TAAAGACATGGGCAAATAC	611953
*galU*-5-F (LA)	5′-GCTGGTCGAATTGCTAACT	611093
*galU*-5-R (LA)	5′-GTATCAATGGCATCAGTTAA	611915
*galU*-6-F (LA)	5′-TTGGCTGGCGTTATCATTT	612396
*galU*-6-R (LA)	5′-GACCGTCATTAAGCATTGTAC	614143
*galU*-7-F (LA)	5′-ATTATAAGGAGGCACTCACCATGGGCAGAAAGTGTATATATA	611190
*galU*-7-R (LA)	5′-CAAAGAAAGCTTGAGCTCTCTAGATTTATTTTTTCGCTTATC	612125
*galU*-8-F (pNZ)	5′-ATTATAAGGAGGCACTCAccatgg	184
*galU*-8-R (pNZ)	5′-tctagaGAGCTCAAGCTTTCTTTG	236

## Data Availability

Data is contained within the article.
